# Calcium Dobesilate Versus Flavonoids for the Treatment of Early Hemorrhoidal Disease: A Randomized Controlled Trial

**DOI:** 10.7759/cureus.9845

**Published:** 2020-08-18

**Authors:** Shabbar H Changazi, Samiullah Bhatti, Ayesha Choudary, Muhammad Naeem Afzal Rajput, Zahid Iqbal, Qamar A Ahmed

**Affiliations:** 1 Surgical Special Unit, Services Hospital, Lahore, PAK; 2 Surgery, Services Hospital, Lahore, PAK; 3 Internal Medicine, Services Hospital, Lahore, PAK; 4 Surgery, Services Institute of Medical Services, Lahore, PAK

**Keywords:** early hemorrhoidal disease, flavonoid therapy, calcium dobesilate therapy

## Abstract

Background and Aim

Early hemorrhoidal disease is usually treated conservatively with fiber diet and medical therapy with flavonoids or calcium dobesilate. The purpose of this study was to compare the efficacy of these two agents in the treatment of early hemorrhoidal disease.

Materials and Methods

Patients having grade I and grade II hemorrhoidal disease were recruited in the study. One group received flavonoid therapy and the other group took calcium dobesilate treatment for three weeks. The symptoms and size of hemorrhoids were then assessed at the fourth week.

Results

In this study, 70.2% of patients were male and 29.8% of patients were female. Of the total patients, 58.65% of patients were below 45 years of age and 41.34% of patients were above 45 years of age. Moreover, 83.65% of patients had grade II hemorrhoids, whereas 16.34% of patients had grade I hemorrhoids; 80.8% of patients showed a decrease in frequency and amount of bleeding after being treated by flavonoids, whereas 67.3% showed a decrease in frequency and amount of bleeding after administration of calcium dobesilate. A decrease in the size of hemorrhoids was seen in 67.3% of patients after treatment with flavonoids and 38.46% after giving calcium dobesilate.

Conclusions

Treatment of early hemorrhoidal with flavonoid therapy was more effective in improving the symptoms of disease as compared to calcium dobesilate treatment.

## Introduction

Hemorrhoids are a common disorder of the perianal region. They are symptomatic enlargement of the anal cushions [1]. Hemorrhoids can be classified into three types depending on the site of formation. Those arising above the dentate line are called internal hemorrhoids, those originating below the dentate line are called external hemorrhoids, and the mixed variety is believed to be present both above and below the dentate line.

Goligher’s classification is used to further classify the internal hemorrhoids into first-, second-, third-, and fourth-degree hemorrhoids depending on the degree of prolapse. Those that bleed but do not prolapse are known as first-degree hemorrhoids, those that prolapse but reduce spontaneously are known as second-degree hemorrhoids, those that require manual replacement are known as third-degree hemorrhoids, and those that are prolapsed and irreducible are known as fourth=degree hemorrhoids [2].

Studies on the prevalence of hemorrhoids are infrequent and have varying results. Johanson and Sonnenberg estimated a prevalence of 4.4% in U.S. adults [3]. Riss et al. in a study of 976 patients attending for colorectal cancer screening found that 38.93% suffered from hemorrhoids [4]. Hemorrhoids are asymptomatic in about 40% of individuals. Symptoms of hemorrhoids vary. The diagnosis of hemorrhoidal disease is based on patient history and clinical examination. Digital rectal examination and proctoscopy form the initial assessment.

The treatment of hemorrhoids varies depending on the degree and severity of symptoms. Lifestyle and dietary modification include increasing the intake of dietary fiber and oral fluids, reducing consumption of fat, having regular exercise, improving anal hygiene, abstaining from both straining and reading in the toilet, and avoiding medication that causes constipation or diarrhea [5,6].

Medications to reduce chronic venous insufficiency include venotonic agents such as flavonoids and calcium dobesilate. Numerous studies have shown the efficacy of flavonoids against early hemorrhoidal disease. In one of the meta-analysis, flavonoids demonstrated a remarkable response in early hemorrhoidal disease, with decreased risk of bleeding by 67%, persistent pain by 65%, and itching by 35% [7]. Calcium dobesilate in conjunction with fiber supplement was shown to be associated with a significant improvement in the inflammation of hemorrhoids [8].

The purpose of this randomized prospective study was to compare the efficacy of these two agents in the treatment of early hemorrhoidal disease.

## Materials and methods

This study was conducted in the Surgical Department of Services Institute of Medical Sciences Lahore, Pakistan, from December 2016 to November 2018. A total of 104 adult patients with first- and second-degree hemorrhoids diagnosed by clinical symptoms and anoscopy examination were included in this study. Ethical approval was taken from Institutional Review Board of the Services Institute of Medical Sciences. Patients of both genders and above 15 years of age with first- and second-degree hemorrhoids were recruited for the study. Patients with third- or fourth-degree hemorrhoids, patients having concurrent fistula or chronic anal fissure or inflammatory bowel disease, patients with previous anorectal surgery, patients having portal hypertension due to cirrhosis or portal vein thrombosis, and patients with a previous history of injection sclerotherapy or band ligation of hemorrhoids were excluded from the study. Written informed consent was taken from all patients. Rectosigmoidoscopy was included in the workup to rule out colorectal cause of symptoms. After initial proctoscopy examination, the patients were randomly allotted to one of the groups. For group A (flavonoids; n = 52) flavonoids (Daflon) tablet 500 mg was given in the following dose: six tablets daily in the first week in BD dose and then four tablets daily in the second week, and, finally, two tablets daily in the third week. The symptoms and size of hemorrhoids were assessed in the fourth week. For group B (calcium dobesilate n=52) calcium dobesilate capsule (500 mg) was given in the dosage of two capsules twice a day for three weeks. The symptoms and size of hemorrhoids were assessed in the fourth week. The patients also received dietary and lifestyle advice. A high-fiber diet was advised to all patients in both groups and they were educated about the importance of fiber (fruits, vegetables, etc.) in healthy nutrition.

Frequency and percentages were calculated for quantitative data. A p-value of 0.05 or less was taken as significant. T-test and chi-square tests were applied in order to compare data of both groups. This work has been reported in line with the Consolidated Standards of Reporting Trials (CONSORT) guidelines and has registered in clinicaltrial.gov (NCT02782455).

## Results

A total of 104 patients with early hemorrhoidal disease (grade I and grade II) were recruited in the study. Out of 104 patients, 73 (70.2%) were males and 31 (29.8%) were females. Out of the total 104 patients, 61 (58.65%) were below 40 years of age and 43 (41.34%) were above 40 years of age. Out of 104 patients, 87 (83.65%) had grade II hemorrhoids, whereas 17 (16.34%) had grade I hemorrhoids, as depicted in Table 1.

**Table 1:** Treatment given according to gender, age group, and grade of hemorrhoidal disease of the patients

Variables		Treatment		Total (n)	Percentage (%)	p-Value
		Flavonoids	Calcium dobesilate			
Gender	Male	38	35	73	70.2	0.520
	Female	14	17	31	29.8	
	Total	52	52	104	100	
Age group	Below 40 years	31	30	61	58.7	0.712
	Above 40 years	21	22	43	41.3	
	Total	52	52	104	100	
Grade	Grade I	11	6	17	16.3	
	Grade II	41	46	87	83.7	0.185
	Total	52	52	104	100	

In the study, 45 (86.53%) patients showed a decrease in frequency of bleeding after being treated by flavonoids, whereas 35 (67.3%) showed a decrease in frequency of bleeding after administration of calcium dobesilate (chi-square test depicted a significance of 0.020). A similar pattern was observed in the patients treated with flavonoids and calcium dobesilate in terms of amount of bleeding. A decrease in the size of hemorrhoids was found in 35 (67.3%) patients after treatment with flavonoids and 20 (38.46%) after giving calcium dobesilate (p = 0.003), as shown in Table 2.

**Table 2:** Response to treatment

Variables		Treatment		Total (n)	Percentage (%)	p-Value
		Flavonoids	Calcium dobesilate			
Decrease in frequency of bleeding	Yes	45	35	80	76.9	0.020
	No	7	17	24	23.1	
	Total	52	52	104	100	
Decrease in amount of bleeding	Yes	45	35	80	76.9	0.020
	No	7	17	24	23.1	
	Total	52	52	104	100	
Decrease in size of hemorrhoids	Yes	35	20	55	52.9	0.003
	No	17	32	49	47.1	
	Total	52	52	104	100	

## Discussion

This study showed that hemorrhoidal disease was more common in males (70.2%) as compared to females (29.8%). Similarly, a study conducted by Ravindranath and Rahul [9] also showed male predominance with a percentage of 66.7%. This pattern of male preponderance was also seen in other studies such as the study conducted by Ali et al. [10], who showed that there was 85% male prevalence, and Khan et al. [11], who also showed similar results with a male prevalence of 75.9%.

In this study, the most frequent age group affected is below 40 years of age (58.6%). These results are in concordance with studies conducted by Ravindranath and Rahul [9] and Ali et al. [10]. On the contrary, in the study conducted by Khan et al. [11], hemorrhoidal disease was more common in the age group above 40 years (58.2%).

In our study, grade II hemorrhoidal disease (83.65%) was more common than grade I disease. This result is differing with the study carried out by Riss et al. [4], which showed that grade I disease (72.9%) is more common than other grades.

In the study, 86.53% of patients responded to flavonoid treatment in terms of decreased frequency and amount of bleeding, whereas 67.3% of patients responded to calcium dobesilate treatment. Furthermore, a decrease in the size of hemorrhoids was seen in 67.3% patients after treatment with flavonoids and 38.46% after giving calcium dobesilate therapy. These results were contradicting with results of the study conducted by Sarabia et al. [12], which showed that there was no significant difference in efficacy of flavonoids (73%) and calcium dobesilate (76%) in hemorrhoidal disease. They stated that both drugs were equally effective and well tolerated [12]. In a study conducted by Alonso‐Coello et al. [7], flavonoids reduced the risk of bleeding and persisting symptoms by 67% and 58%, respectively. The study conducted by Patel et al. [13] showed that after six weeks of calcium dobesilate therapy, cessation of rectal bleeding plus lack of severe anitis occur in 48 patients, with a success rate of 81.35%. As per our study results, the efficacy of flavonoid therapy is superior to that of calcium dobesilate. However, more studies with larger samples are required to evaluate the efficacy of these drugs.

## Conclusions

It can be concluded that flavonoid therapy was more effective in improving the symptoms of disease in terms of frequency of bleeding, amount of bleeding, and size of hemorrhoids as compared to calcium dobesilate treatment in patients with early hemorrhoidal disease.
